# Research highlights for issue 7: the evolution of invasiveness

**DOI:** 10.1111/eva.12292

**Published:** 2015-07-16

**Authors:** Britt Koskella

With continually increasing global connectivity and wholesale change of landscapes, the need to understand and control the spread of invasive species has never been greater. To do so requires insight into the factors that allow particular species to become invasive to begin with as well as into the evolutionary trajectory those invasive populations follow after establishment in new ranges.

One of the more prominent hypotheses explaining the success of invasive species relies on the idea that populations can disperse away from their natural enemies, and thus grow without the constraints of antagonist-mediated control. Recent work by Tiantian Lin et al. builds on this idea by demonstrating parallel evolution of invasive plant populations across three geographically distinct regions towards decreased investment in defense and increased competitive ability. By comparing the competitive ability of native versus invasive genotypes of Common ragwort in either the absence of herbivores or in the presence of either a specialist or a generalist herbivore, the authors were able to show that invasive genotypes outcompete their native conspecifics in both the absence of herbivores and presence of the generalist herbivore, but have lowered defenses against the specialist herbivore (Lin et al. 2015).

Another prominent idea in the field is that increased phenotypic plasticity of invasive species underlies their ability to successfully invade new habitats. Despite being a longstanding point of discussion, however, this idea has proven difficult to demonstrate empirically. New work by Russel Lande clarifies that quantitative genetic theory does not necessarily predict increased, long-term plasticity of invasive species. Instead, he argues that the outcome will depend on the optimal phenotype in the native versus colonized environments, as well as the predictability of each environment and the cost of plasticity (Lande 2015). Furthermore, he suggests that increased plasticity will often be hard to observe given the short-lived nature of the associated fitness advantage once successful colonization and adaptation have occurred. Indeed, recent evidence from the invasive Japanese stiltgrass suggests rapid adaptation to local abiotic conditions (Ziska et al. 2015), suggesting that any initial plasticity was likely transient.

In line with this idea, a recent hypothesis formalized by Qiao Huang et al. suggests that invasive species may initially be under selection for increased adaptive plasticity due to their release from the stress of their native habitat. The authors develop a model to argue that release from local stressors such as natural enemies would both increase the benefits of plasticity and reduce potential costs, for example through the need to allocate resources to traits other than maintaining the ability to be plastic (Huang et al. 2015). Again, this increased plasticity is likely to be transient as the invasive species adapts to its local environment and, reciprocally, the local biotic environment responds to the presence of the invasive species.

A contrasting hypothesis for invasiveness is the idea that introduced species succeed if/when they are able to occupy an open niche in the introduced range. Katrina Dlugosch et al. recently set out to test this hypothesis using the highly invasive yellow starthistle plant, which has spread far outside its native Eurasian range into Australia, Argentina, Chile, and the USA. Using a greenhouse experiment on invasive populations from Californian grasslands, the authors demonstrate that the plant has evolved an increased dependence of water allowing it to reach a higher fitness (Dlugosch et al. 2015). This niche exploitation is possible due to local declines in resident competitors, indicating that loss of biodiversity can open new niches and promote invasion by non-native species.

Finally, a recent hypothesis put forward by Jessica Stapley et al. suggests that invasiveness may be driven in part by increased activity of transposable elements allowing rapid adaptation despite the small size (and thus low genetic variation) of most founding populations. The authors describe how environmental and genomic perturbations typical of invasion may favor activation of transposable elements, and therefore increase the rate of introduction of potentially beneficial alleles (Stapley et al. 2015).

## References

[b1] Dlugosch KM, Cang FA, Barker BS, Andonian K, Swope SM, Rieseberg LH (2015). Evolution of invasiveness through increased resource use in a vacant niche. Nature Plants.

[b2] Huang QQ, Pan XY, Fan ZW, Peng SL (2015). Stress relief may promote the evolution of greater phenotypic plasticity in exotic invasive species: a hypothesis. Ecology and Evolution.

[b3] Lande R (2015). Evolution of phenotypic plasticity in colonizing species. Molecular Ecology.

[b4] Lin T, Klinkhamer PG, Vrieling K (2015). Parallel evolution in an invasive plant: effect of herbivores on competitive ability and regrowth of *Jacobaea vulgaris*. Ecology Letters.

[b5] Stapley J, Santure AW, Dennis SR (2015). Transposable elements as agents of rapid adaptation may explain the genetic paradox of invasive species. Molecular Ecology.

[b6] Ziska LH, Tomecek MB, Valerio M, Thompson JP (2015). Evidence for recent evolution in an invasive species, *Microstegium vimineum*, Japanese stiltgrass. Weed Research.

